# Research on Guidance Methods of Digital Twin Robotic Arms Based on User Interaction Experience Quantification

**DOI:** 10.3390/s23177602

**Published:** 2023-09-01

**Authors:** Wenyu Wu, Mingrui Li, Jincheng Hu, Shuwei Zhu, Chengqi Xue

**Affiliations:** School of Mechanical Engineering, Southeast University, Nanjing 211100, China; 220204819@seu.edu.cn (J.H.); 230229258@seu.edu.cn (S.Z.); ipd_xcq@seu.edu.cn (C.X.)

**Keywords:** interactive experience, digital twin robotic arm, virtual reality, trajectory input, guidance methods

## Abstract

The evolution of the manufacturing sector coupled with advancements in digital twin technology has precipitated the extensive integration of digital twin robotic arms within the industrial domain. Notwithstanding this trend, there exists a paucity of studies examining the interaction of these robotic arms in virtual reality (VR) contexts from the user’s standpoint. This paper delves into the virtual interaction of digital twin robotic arms by concentrating on effective guidance methodologies for the input of their target motion trajectories. Such a focus is pivotal to optimize input precision and efficiency, thus contributing to research on the virtual interaction interfaces of these robotic arms. During empirical evaluations, metrics related to human–machine interaction, such as objective operational efficiency, precision, and subjective workload, were meticulously quantified. Moreover, the influence of disparate guidance methods on the interaction experience of digital twin robotic arms and their corresponding scenarios was investigated. Consequent findings offer pivotal insights regarding the efficacy of these guidance methods across various scenarios, thereby serving as an invaluable guide for future endeavors aiming to bolster interactive experiences in devices akin to digital twin robotic arms.

## 1. Introduction

Advancements in technology have fostered the widespread adoption of distributed sensor networks, machine learning, comprehensive data mining, and virtual simulation visualization within the manufacturing sector [1]. Smart industrial robotic arms, showcasing capabilities such as system autonomous perception, autonomous decision-making, adaptive cycling, self-learning, and human–machine collaboration, have seen accelerated growth. Digital twin technology, pivotal in revolutionizing intelligent manufacturing, can bolster predictive maintenance, facilitate real-time data analytics and decision-making support, and expedite prototyping and testing. Such robotic arms assist engineers in simulating the design, manufacturing, and operation phases with heightened precision, thereby curtailing research and operational expenses while augmenting efficiency and accuracy.

**Figure 1:**
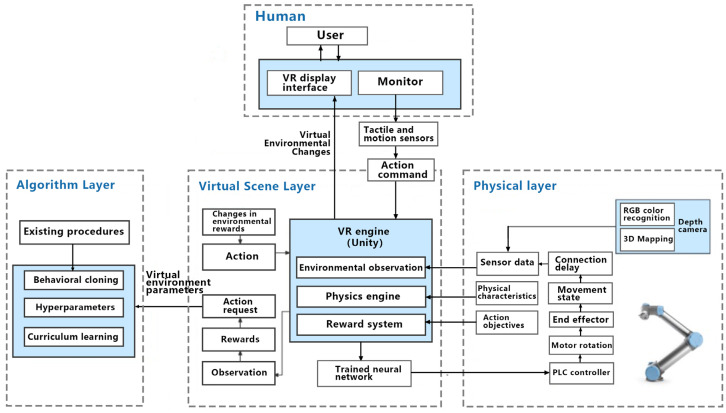
Technology roadmap of virtual reality interaction design in digital twin scene.

In the present landscape, the majority of interactive platforms, inclusive of digital twin interfaces, predominantly operate in two dimensions. However, the evolution of information technology, paired with the continuous upgrades of devices, has propelled virtual reality to offer a multimodal perception in human–computer interactions. Marrying digital twin interactivity with virtual reality environments aligns the interaction more congruently with user intuition [2], thereby enhancing the utility and efficacy of digital twin systems [3]. Specifically, within the domain of industrial robotic arms, remote operation and visualization of these digital twin robotic arms become feasible. The technological trajectory of virtual reality interaction within the digital twin context is delineated in Figure 1.

This project revolves around the exploration of guidance methodologies for inputting target motion trajectories of digital twin robotic arms, anchored on the quantification of user interaction experience. It constitutes a segment of the broader research on the design of interactive interfaces for robotic arms. The scope of the work encompasses theoretical investigations, digital twin robotic arm task execution processes, user requirements studies, and interactive experimentations. Given that robotic arm operations frequently involve high-precision movements, maintaining accuracy is paramount for ensuring quality task outcomes. Precise motions of digital twin robotic arms demand operator inputs via an interactive interface, enabling the adjustment of the robotic arm’s movement and workflow. When inputting motion trajectories, the design of the interactive interface should guide users through the process, facilitating accurate operations and aiming to ensure the precision of the target motion trajectory. This precision, in turn, allows the robotic arms to execute tasks like cutting, drilling, or grasping with enhanced accuracy. Beyond its direct implications for task accuracy, this guidance approach—being integral to the design of the digital twin interaction interface—profoundly influences user operation experience and ranks as a top user requirement. This research is rooted in human–computer interaction, emphasizing the influence of guidance methods on user task accuracy and holistic interaction experience. The study then translates these influences into both objective and subjective metrics, highlighting the unique features of interaction methodologies. Consequently, understanding this interaction dynamic is pivotal for operating the robotic arm’s digital twin interface and offers invaluable insights for the conceptualization of analogous digital twin devices and systems.

## 2. Research Status

### 2.1. Robotic Arms Based on Digital Twins

Gaurav Garg et al. [4] tackled the primary challenges associated with contemporary robot programming methodologies, such as the drafting and alteration of code for robot trajectories. They further explored the simplification of these codes via digital twins and scrutinized the trajectory replication between digital and physical robots, leveraging both digital twin and virtual reality technologies. Ahm Shamsuzzoha [5] assessed the employment of virtual reality platforms, postulating that VR platforms rooted in robotic arms offer an immersive human–machine interface, holding potential as instruments for industrial training and maintenance services. This study also showcased the application of virtual reality technology in the management and maintenance of robotic arms. Marius Matulis et al. [6] delved into the processes of creating and instructing robotic arm digital twins, executing AI training in virtual environments, and extrapolating this simulation-based learning to tangible space.

Torbjorn Moi et al. [7] introduced an innovative approach to monitor the state of small articulated cranes using digital twins. They implemented an inverse method to estimate weight and its force vector orientation, drawing upon data from physical strain gauges. Aitor Ardanza [8] unveiled a fully operational hardware coupled with a groundbreaking software architecture, designed to construct a versatile advanced HMI interface, aiming to equip machine operators with dynamic and pertinent data. Cai [9] suggested a technique employing augmented reality to streamline the conveyance of layout data between reconfigurable additive manufacturing (AM) systems, inclusive of robotic arms, and their analogous digital twins, targeting tool path strategizing and simulation. Zong [10] integrated digital twin technology into workshop operational simulations and monitoring, furnishing data-driven insights for workshop production evaluation.

### 2.2. Digital Twin Interaction Design

Scholars are committed to research on the perception and recognition of objects in digital twins, which involves fundamental technologies in digital twins. Xiong et al. [11] proposed a deep supervised subspace learning method to assist robots in perceiving the properties of objects during non-contact interaction. By utilizing the low noise and fast response advantages of non-contact sensors, non-contact feature information is extracted to detect cross modal information and infer the material properties of objects. Experiments were conducted to verify the effectiveness of the method. Xiong et al. [12] proposed a novel few-shot learning with coupled dictionary learning (FSL-CDL) framework to address the issue of poor object perception using spectral measurements under few-shot learning due to insufficient training samples. The accuracy was verified through experiments. Generally speaking, most scholars attempt to solve abstract tactile target recognition problems with a single feature extraction method. Xiong et al. [13] fused measurements based on a new sparse encoding model to avoid these defects. This model not only preserves the original multimodal information but also transforms the original data into a shared feature space.

At the level of digital twin interaction, Enes Yigitbas et al. [14] introduced a methodology to augment the human-in-the-loop system by integrating digital twins with VR interfaces. To facilitate human participation in decision-making, two distinct “people in the loop” strategies—program control and VR interaction control—were assessed and implemented within a VR context. Jesper Rask Lykke et al. [15] posited that neglecting an object’s weight and moment of inertia could diminish user immersion. In response, they advanced a high-fidelity interaction approach for virtual objects, enabling actions like picking, processing, swinging, and throwing based on attributes such as weight, dimensions, and load capacity. Kim et al. [16] designed a user interface predicated on gaze pointers, tailoring interactions for mobile platform virtual reality settings. The devised 3D interactive content for mobile platforms encompassing four primary design elements: field of view, feedback mechanisms, multi-dimensional data relay, and background coloration. The study also probed potential adverse psychological impacts, investigating issues like VR sickness, fatigue, and interaction difficulties. Kenan Bektaş et al. [17] introduced an innovative interface named the Limbic Chair. The efficacy of the Limbic Chair was showcased in two VR contexts: urban navigation and flight simulation. Comparative studies between the Limbic Chair and traditional game controllers were executed, employing metrics like performance, head movement, body oscillation, and standard questionnaires to draw inferences about usability, workload, and simulator sickness. Lastly, Jan Niklas Voigt Antons et al. [18] embarked on an exploration of user experiences concerning various VR interaction modalities, either via controllers or hand tracking, and evaluated them using both the System Availability Scale (SUS) and Self-Assessment Model (SAM).

### 2.3. Summary of Research Status

Currently, research on digital twin systems predominantly centers on the development of virtual and physical layers. However, the significance of multimodal interaction in digital twin applications is sometimes undervalued. In terms of virtual interaction, many studies on digital twins concentrate on the interaction technologies rooted in natural gestures and tactile feedback. Simultaneously, there exists a marked need for increased research on the design and refinement of the comprehensive interaction interface.

## 3. Completed Work

### 3.1. Theoretical Research

The paradigm of the digital twin interaction interface has been extensively researched to amplify the efficiency of information transmission within the interface. This encompasses the implementation-centered, metaphorical, and idiomatic paradigms.

Studies have delved into the information architecture of digital twin robotic arms to ensure that users can effortlessly locate the desired information and execute tasks [19]. Such architectures are categorized into hierarchical, sequential, mesh, and matrix models.

Research on the interactive interface of digital twin robotic arms has been comprehensive, encompassing various interface forms. This includes control equipment such as handles and HMDs, dashboard styles, modular and situational layouts, and elemental aspects like range, depth, color, and icons.

### 3.2. Task Execution Process of Digital Twin Robotic Arms

The procedure for the interaction task associated with robotic arm operation has been delineated, encompassing steps such as system login, control mode selection, motion parameter configuration, motion path planning, motion initiation, real-time monitoring and adjustments, and eventual cessation with data preservation.

Furthermore, additional systemic tasks have been identified, comprising task management, model oversight, data visualization and querying, alarm and malfunction resolution, and user administration. Explicitly, these processes involve task formulation, editing, and elimination; data import/export; model uploading, downloading, updating, and management; synchronized display of robotic arm status with its digital twin; real-time data chart visualization; historical data retrieval; alarm notifications; fault diagnosis; user login; privilege configuration; and user data alterations. Collectively, these processes bolster the system’s comprehensive functionality and efficiency.

### 3.3. Research on User Demands

The determination of demand priority can be achieved by administering a questionnaire survey and subsequently analyzing the non-linear relationship between product performance and user satisfaction through the KANO model [20]. A total of thirty-seven valid questionnaires were gathered. The content of the questionnaire can be found in Appendix A, while the specific interaction demands investigated are detailed in Table 1.

The questionnaire was designed to evaluate nine specific needs. Respondents were prompted to rate each need based on the following dimensions: “I really like it”, “It should be”, “Indifferent”, “Settle for”, and “I really do not like it”. The aggregated scoring outcomes are presented in Table 2. For the KANO model’s feature attributes, the numerical calculation method involves summing the values of A, O, M, I, R, and Q from the table and then dividing by the product of 25 and 44.

As shown in Table 3, a summary table of all demand characteristics is provided, where Better value = (A + O)/(A + O + M + I) and Worse value = (M + O)/(A + O + M + I).

**Figure 2:**
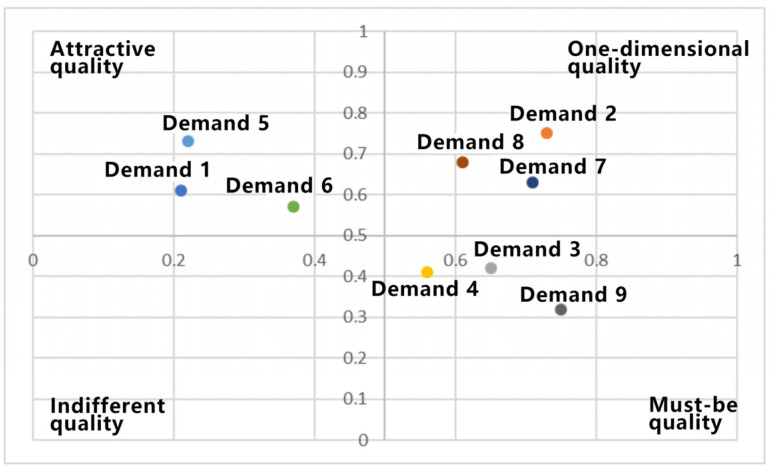
Better–Worse coefficient analysis.

The above interaction demands are divided and prioritized using a four-quadrant diagram, as shown in Figure 2.

Demands characterized by the “must be quality” tendency take precedence over those with the “one-dimensional quality” tendency. Subsequently, those demands are given priority over those reflecting the “attractive quality” tendency. Further ranking is based on better value. Thus, the prioritized order of the stated demands is as follows: robotic arm interaction guidance methods, reasonable interaction feedback forms, cognitive information management functions, logical interface layout, structured information architecture, visual representation of information, effective interactive navigation, task-specific interaction interface presentation, and optimal sensory experience.

## 4. Research on Trajectory Planning Guidance Method for Digital Twin Robotic Arms

### 4.1. Experimental Purpose

**Figure 3:**
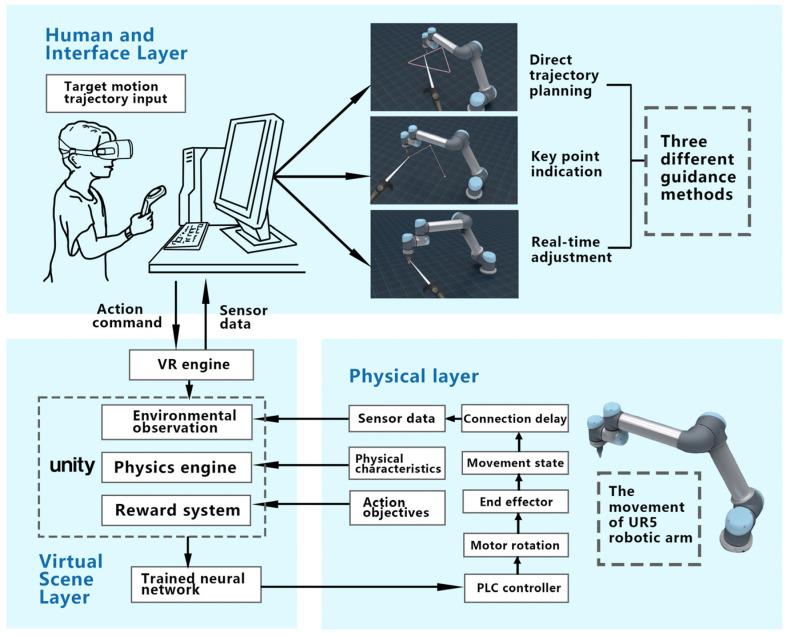
The process of operators planning the motion of real robotic arms through a digital twin system.

As delineated in the earlier “completed work” section, during task configuration, the operator is required to specify the desired motion trajectory of the robotic arm. This human–machine interaction necessitates the system to offer guidance and support throughout its execution. This is twofold: Primarily, it ensures the accuracy of the intended motion and thereby ensures task completion quality. Additionally, user needs assessment indicates that interactive guidance is the paramount demand. Consequently, the guidance mode for the digital twin manipulator’s target motion trajectory emerges as a crucial aspect of this system’s interaction research. This also serves as a benchmark for digital twin interaction designs and research on analogous equipment. The operator at the control end inputs this process. Subsequently, the finalized trajectory is transposed onto the motion of the actual robotic arm through apparatuses like virtual reality engines, neural networks, and PLC controllers. The detailed procedural logic is depicted in Figure 3.

In the experiment, the input guidance methods for the target motion trajectory encompass direct trajectory planning, key point indication, and real-time adjustments, all intended to enhance user experience. These guidance methods each have distinct characteristics, presenting both advantages and disadvantages in specific task contexts. Users, in turn, exhibit varied preferences towards these interaction techniques, leading to differential work efficiencies. Utilizing a data-driven methodology, this article seeks to capture these nuances in preferences, experiences, and efficiencies to facilitate a comprehensive analysis, discussion, and interpretation of the experiments. Consequently, the experiment’s objective is to contrast the variances in task completion efficiency and interaction among the diverse guidance methods, especially as applied to robotic arm tasks, by quantifying the interactive operational experience.

### 4.2. Experimental Design

#### 4.2.1. Experimental Variables

This experiment identifies two independent variables: path planning difficulty, classified into straight segment paths and irregular arc paths, and guidance methodologies for target motion input, namely, direct trajectory planning, key point indication, and real-time adjustment.

Direct trajectory planning involves the user sketching the anticipated motion path directly within a virtual environment using linear and curve tools. This guidance allows users to input directly via the handle controller on the interface.

In the key point indication method, the motion planning is guided by pinpointing crucial points on the robotic arm’s motion path. Here, users designate a series of these key points on the interface and the system autonomously produces a smooth motion path based on these points. Users can continuously add, delete, or adjust these points until satisfied. Upon finalizing the path planning, the robotic arm will traverse each key point sequentially along the pre-established path.

The real-time adjustment method offers the possibility for users to perpetually refine the path during the robotic arm’s motion. This guidance allows users to control the robotic arm’s live motion through the interface, adjusting the trajectory on-the-go to meet task prerequisites. Throughout this real-time modification, participants manipulate the handle to alter the end effector’s trajectory of the robotic arm. It necessitates vigilant monitoring of the robotic arm’s actual motion to ensure adherence to the pre-set path. This method essentially synchronizes target motion trajectory input with the arm’s movement in real-time.

Furthermore, user interaction experience is bifurcated into objective and subjective dimensions, which are quantified based on the experimental prerequisites and device operational attributes. Objective metrics predominantly concern operation outcomes, specifically operational efficiency data. Given that the robotic arm’s movement epitomizes the experiment’s primary objective, path deviation serves as a suitable objective metric. Concurrently, operation duration, symbolizing “efficiency”, should also be incorporated. Subjective metrics capture users’ sentiments during the interaction. As operating a robotic arm is task-centric, the user’s perception chiefly mirrors the perceived task load. Hence, this load is interpreted as subjective data using the Subjective Workload Index (NASA-TLX), comprising six facets: mental demands, physical demands, temporal demands, performance, effort, and frustration. This ensures a comprehensive, multi-dimensional evaluation of the interactive experience.

#### 4.2.2. Experimental Process

Initially, participants were briefed on the experiment’s objectives and the guidance methods for target motion trajectory. Subsequently, they acquainted themselves with the environment and practiced operating the robotic arm using the three path planning guidance methods.

During the experiment, participants employed VR devices to execute three trajectory planning and guidance tasks, presented in a randomized sequence. Each guidance method encompassed two tasks: navigating the end effector along an equilateral triangle path and traversing an irregular arc path, culminating in six tasks in total. Metrics such as task completion time and path deviation indicators during the robotic arm’s movement were meticulously recorded.

**Figure 4:**
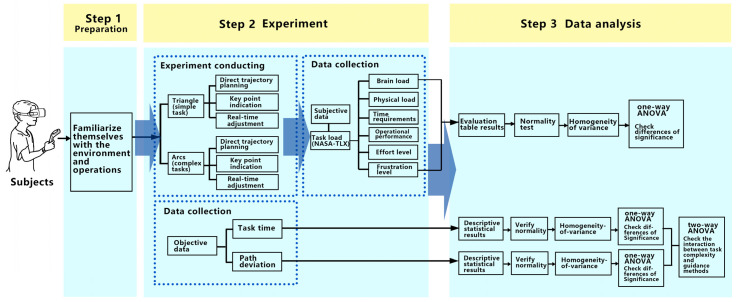
Experimental process.

The experiment resulted in 20 users undertaking 6 tasks each, leading to a cumulative 120 interactive tasks. Post-experiment, participants completed a subjective questionnaire and furnished personal details. Interview sessions were also held, during which the experimenter documented participants’ experiences, inclinations, and feedback. The stepwise experimental process is delineated in Figure 4.

#### 4.2.3. Experimental Implementation

1. Experimental Participants

A total of twenty participants were enrolled, comprising 13 males and 7 females, with ages ranging from 22 to 27. All were students at South-east University and had some proficiency in computer operation. Notably, six participants had prior experience with virtual reality interaction.

2. Experimental Environment

The laboratory provided a serene and interruption-free environment, maintaining optimal temperature, humidity, and illumination conditions.

Hardware Specifications:

Processor—Intel Core i7;

Memory—32 GB RAM;

Graphics card—NVIDIA GeForce GTX 1660 Ti.

The hardware used for the experiment was the HTC VIVE Pro Eye. Specifications of the head-mounted display include the following:

Refresh rate—90 Hz;

Resolution—2880 × 1600;

Screen type—OLED;

Field of view—110°.

Furthermore, the virtual environment was rendered by a PC capable of supporting multiple video interfaces.

Software Configuration:

The software was built on the Unity 2018.2.10fl (64-bit) platform, integrated with the Steam VR SDK, optimized for the HTC VIVE virtual reality devices. User data from the experiments were archived within the Controller Recorder.

**Figure 5:**
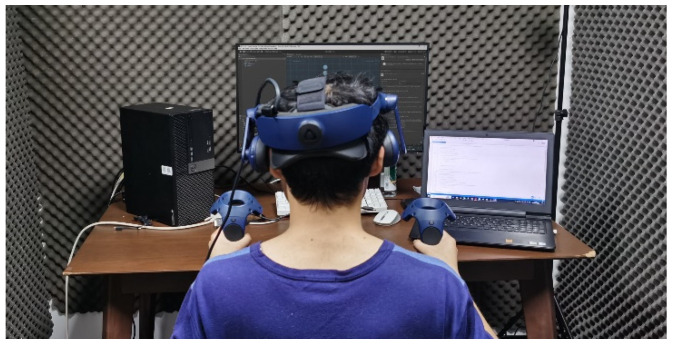
Experimental environment.

The experimental environment is depicted in Figure 5.

3. Task Settings

**Figure 6:**
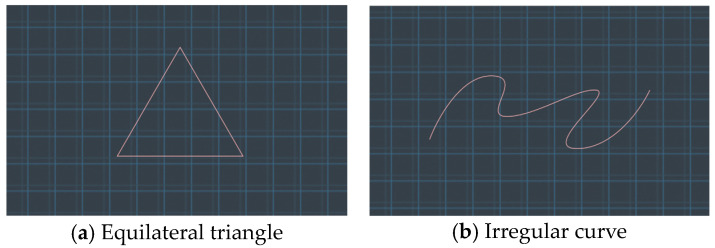
Two types of trajectory tasks.

Before commencement, the desired motion trajectory for the end effector was visualized on the screen. Participants were instructed to use three guiding methods to traverse two distinct trajectories: an equilateral triangle (side length: 1 m) and a curved pathway (total length: 3 m), as depicted in Figure 6. Subsequently, the robotic arm retraced the path charted by the participants, initiating from the start and culminating at the terminus. For experimental simplicity, the robotic arm’s movement was standardized at a steady pace of 1 m/s, overlooking joint influence and motor acceleration durations. Upon task culmination, metrics like completion time and the robotic arm’s path deviations during its course were systematically recorded.

4. Task Execution

The duration required to complete each task during the experiment is automatically documented and archived within the SQLite database. Path deviations are determined by comparing the distance between individual points on the entered motion trajectory and their nearest points on the anticipated target trajectory. These deviations, indicative of accuracy disparities, are subsequently collected and scrutinized within the Unity environment using pertinent algorithms.

### 4.3. Data Analysis

#### 4.3.1. Objective Data

1. Task Duration

The objective data of the experimental participants are recorded by the Controller Recorder in the XR Interaction Toolkit.

a. Data Analysis for “Triangle” Task.

Table 4 shows the descriptive statistical results of the three guidance methods and task completion time. The direct trajectory planning task has the shortest completion time, followed by the key point and real-time adjustment methods.

Initially, the data distribution’s normality was assessed. As delineated in Table 5, the *p*-value for both direct trajectory planning and real-time adjustment, when subjected to the Kolmogorov–Smirnov test, is less than 0.05, albeit proximate to this threshold. Given the limited sample size, asserting the normality of the data can be challenging, especially in light of stringent normality test criteria. Consequently, utilizing normal or Q-Q plots is advised for normality verification. If the data exhibit attributes consistent with normality, they can be deemed to follow a normal distribution.

**Figure 7:**
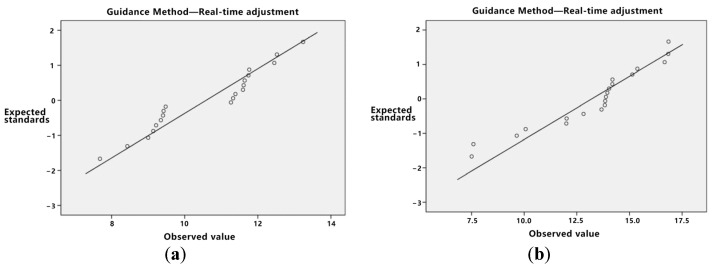
Standard Q-Q diagram. (**a**) Standard Q-Q diagram for direct trajectory planning. (**b**) Standard Q-Q diagram for real-time adjustment.

As depicted in Figure 7, both the values of direct trajectory planning and real-time adjustment exhibit characteristics consistent with a normal distribution. Additionally, the key point indication method also conforms to normal distribution traits. Further analysis, as evidenced by the homogeneity of variance test presented in Table 6 with a *p*-value greater than 0.05, confirms the homogeneity of variance of the data, rendering them suitable for variance analysis.

As presented in Table 7, the *p*-value associated with the guidance method is 0.001. This value is substantially below the 0.05 threshold, signifying notable differences in the task completion times across the three guidance methods.

**Figure 8:**
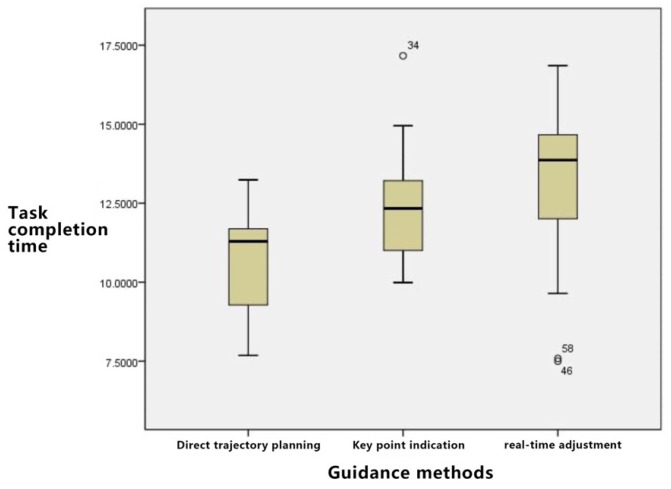
Task completion time block diagram for three guidance methods in “Triangle” Task.

As depicted in Figure 8:

Time Consumption Analysis: The order of time consumption, from least to most, is direct trajectory planning, key point indication, and real-time adjustment. Notably, variances exist within each group due to the individual skills and experience of the operators; however, the disparity between groups remains manageable.

b. Data Analysis for “Curve” Task:

The descriptive statistical results displayed in Table 8 for the irregular curve path tasks reveal the following: direct trajectory planning consumes the most time, succeeded by key point indication and then real-time adjustments.

Firstly, normality tests and homogeneity of variance tests are conducted.

**Figure 9:**
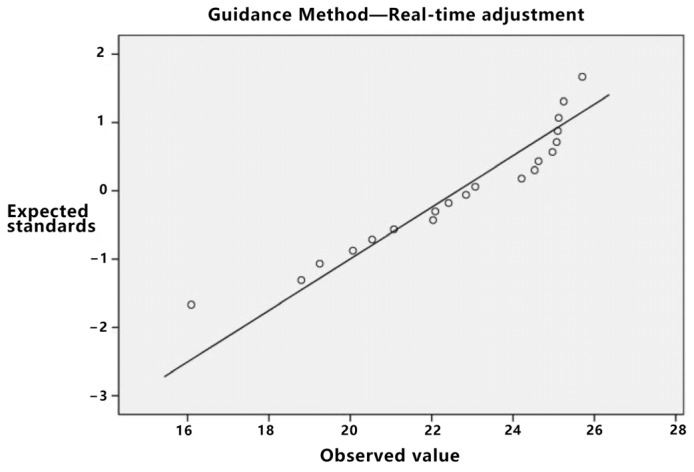
Direct trajectory planning standard Q-Q diagram.

Based on Table 9, the *p*-value for direct trajectory planning stands at 0.048, which is less than 0.05. However, Figure 9’s Q-Q diagram substantiates its adherence to a normal distribution. Subsequently, the homogeneity of variance of the data was examined, resulting in a *p*-value less than 0.05. This suggests that the data do not conform to the homogeneity of variance prerequisite and, hence, are unsuitable for a variance analysis. Given the normal distribution of the data, Welch’s analysis of variance was employed. Table 10, where *p* is less than 0.05, confirms a significant discrepancy among the three guidance methods.

**Figure 10:**
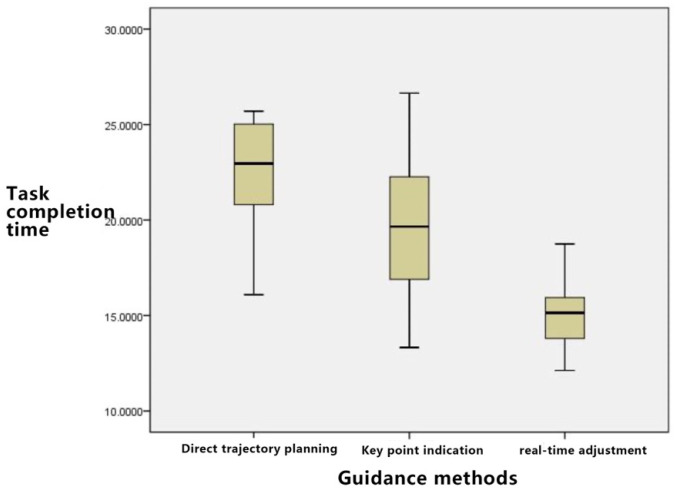
Task completion time block diagram for three guidance methods in “Curve” Task.

As depicted in Figure 10, for the irregular curve path task, the time required follows the sequence: direct trajectory planning > key point indication > real-time adjustment. The direct trajectory planning method demands a sophisticated skill set from the user. While the key point indication consumes some time for point-setting operations, its implementation remains straightforward. For irregular curves, the real-time adjustment feature emerges as particularly apt.

Subsequently, a two-way analysis of variance is utilized to examine the interaction between task complexity and trajectory guidance methods.

Table 11 illustrates a significant interaction between the guidance methods and task complexity with respect to task completion time.

2. Path Deviation

a. Data Analysis for the “Triangle” Task

As presented in Table 12, when examining the path deviations under the “triangle” task for the three trajectory guidance methods, it is observed that direct trajectory planning has the least deviation. This is subsequently followed by key point indication and, lastly, real-time adjustment.

Based on Appendix B, the data meet the criteria for the normality test. Furthermore, the homogeneity of variance test reveals that all *p*-values for the data exceed 0.05, permitting the use of analysis of variance. As detailed in Table 13, a *p*-value of less than 0.05 signifies significant differences among the three trajectory guidance methods in the triangular path task.

**Figure 11:**
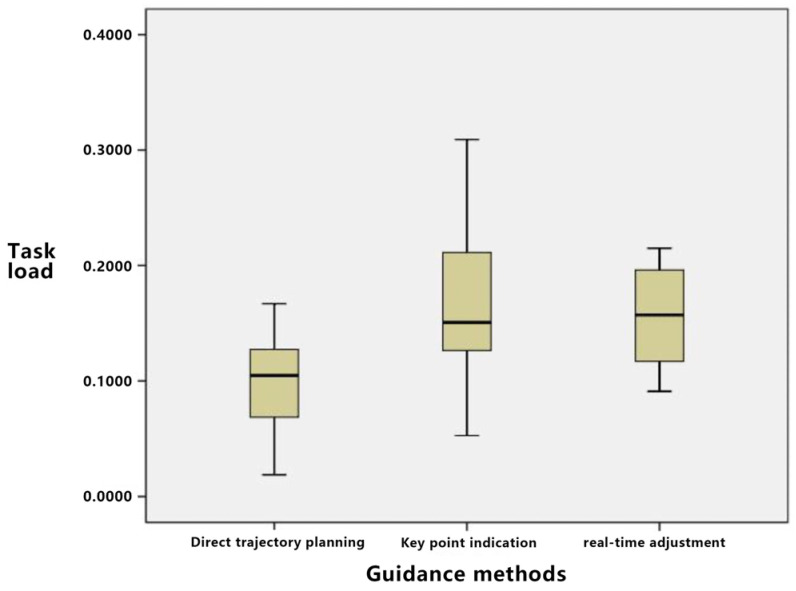
Block diagram of path deviation under simple tasks.

As depicted in Figure 11, for straightforward tasks composed primarily of linear patterns, such as triangles, direct trajectory planning yields the least path deviation and is intuitive for operators. Although the key point indication strategy is less effective than direct trajectory planning, an increase in the number of key points enhances path accuracy at the potential cost of elevated cognitive load. The real-time adjustment method, on the other hand, demonstrates a notable path deviation.

b. Data Analysis for the “Curve” Task

Table 14 offers a detailed statistical account of the path deviations associated with the three guidance methods during the “curve” task. Preliminary observations suggest that direct trajectory planning exhibits the most pronounced path deviation, with real-time adjustment and key point guidance following in succession.

From a statistical standpoint, the experimental data were rigorously analyzed. The aforementioned data underwent both normality and homogeneity of variance assessments, necessitating further elaboration. Consequently, a one-way ANOVA can be executed. As indicated in Table 15, a significant disparity in path deviation is observed among the three guidance methods when applied to complex tasks.

**Figure 12:**
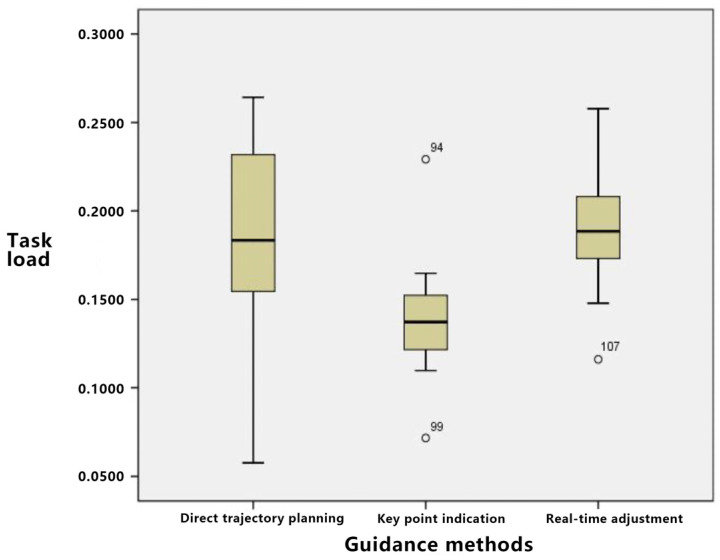
Block diagram of path deviation under complex tasks.

Figure 12 illustrates that for tasks of higher complexity, such as those involving “curves”, direct trajectory planning exhibited significant path deviations. Among the methods, key point indication showcased the least path deviation, with the intrinsic characteristics of its key point settings ensuring minimal deviation. The real-time adjustment method also demonstrated low path deviation, rendering it particularly appropriate for curved paths.

A two-way ANOVA was employed to analyze the interaction between task complexity and guidance methods concerning the input of target motion trajectories. As deduced from Table 16, the interplay between guidance methods and task complexity profoundly influences task deviation.

#### 4.3.2. Subjective Data

Data related to task load were gathered as subjective measurements. The assessment of this task load was facilitated using the Subjective Workload Index (NASA-TLX). This index encompasses factors such as mental demand, physical demand, temporal demand, performance, effort, and frustration level. Since the contributing factors of the task load in this study were not assigned weights through specific evaluation methods, the comprehensive load is represented by the average sum of all these factors. Table 17 delineates the corresponding statistical results.

**Figure 13:**
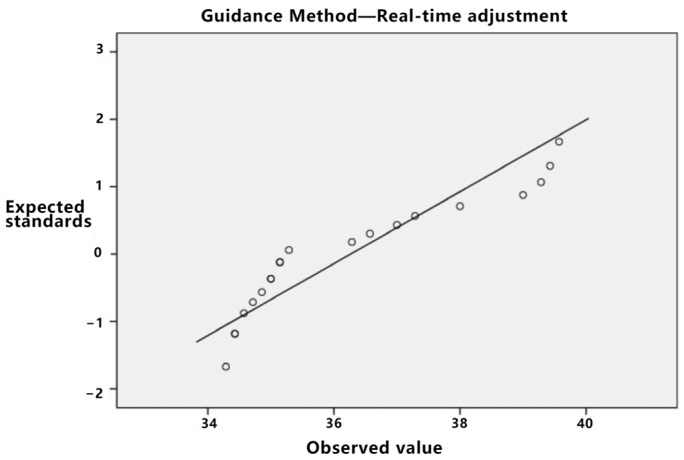
Standard Q-Q diagram of task load under real-time adjustment.

The pertinent data were subjected to statistical analysis. Initially, a normality test was executed. As depicted in Table 18, the *p*-value for the real-time adjustment is *p* = 0.02, which is less than 0.05. Based on the standard Q-Q plot illustrated in Figure 13, it can be inferred that the real-time adjustment method conforms to a normal distribution.

According to Table 19, the *p*-values are all greater than 0.05; so, the data meet the homogeneity of variance and can be analyzed for variance.

A one-way ANOVA was conducted on the task load of the three guidance methods, and the results are shown in Table 20. The *p*-value is less than 0.05, indicating significant differences in task load among the three guidance methods.

**Figure 14:**
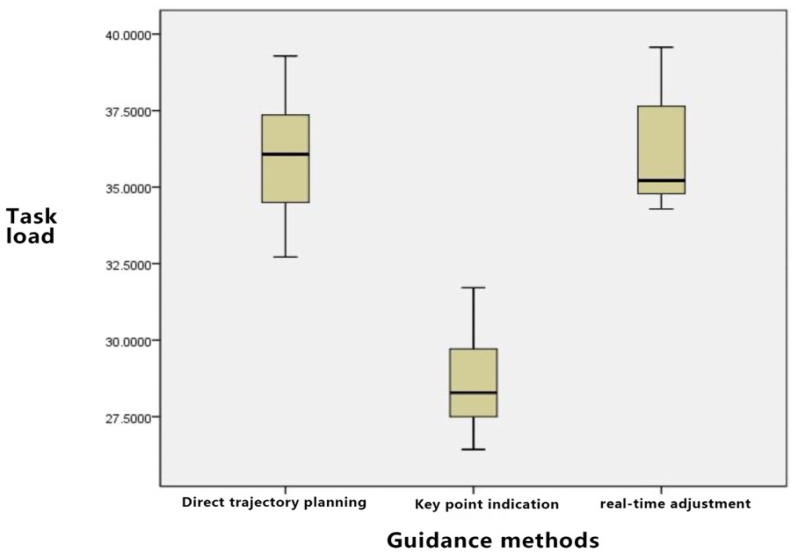
Block diagram of guidance method on task load.

As shown in Figure 14, in terms of mental power consumed, direct trajectory planning has a higher demand because users need to consider the entire trajectory. Key point indication only needs to focus on key point settings, with relatively low brain power consumed. The brain power consumed for real-time adjustment is between those of the first two.

The quantitative results pertaining to subjective interaction experience provide grounds for the following conclusions:

Regarding physical power needs, there is a negligible difference among the three guidance methods, given that operations predominantly hinge on the interface and equipment utilized.

When assessing time requirements, direct trajectory planning might necessitate an extended period for full path planning. Conversely, key point indication proves more expedient as it mandates the setting of only the key points. The time requisite for real-time adjustment is contingent upon user modification frequency, with no pronounced disparity compared to direct trajectory planning.

Considering operational performance, direct trajectory planning tends to demonstrate superior performance for simpler paths but possibly underperforms with more intricate paths. Key point indication facilitates commendable operational performance, given that determining key points curtails complexity. The performance discrepancy between real-time adjustment and direct trajectory planning remains marginal.

In relation to effort level, direct trajectory planning mandates substantial initial effort from users to devise the complete path. Its effort magnitude is intermediate, bracketed by key point indication and real-time adjustment. Key point indication demands the least effort, as focus predominantly lies on key point configuration. Conversely, real-time adjustment is the most labor-intensive, necessitating on-the-fly modifications.

Concerning frustration levels, for convoluted paths, users employing direct trajectory planning might experience pronounced frustration, given the challenge of achieving an impeccable path in a single attempt. Its frustration index is intermediate, nestled between key point indication and real-time adjustment. Key point indication elicits relatively minimal frustration, enabling users to iteratively refine key points for path optimization. Real-time adjustment, demanding incessant modifications, might culminate in the pinnacle of user frustration due to the constant vigilance and error susceptibility.

Evaluating the holistic task load, key point indication emerges as the most benign, with marginal variation discerned between direct trajectory planning and real-time adjustment.

### 4.4. Experimental Summary

In this study, VR technology and equipment were employed to investigate three guidance methods for robotic arm trajectory planning: direct trajectory planning, key point indication, and real-time adjustment. Performance metrics, including execution time, path deviation, and task load, were scrutinized for both simple tasks (equilateral triangles) and complex tasks (irregular curves).

The analyses pertaining to execution time and path deviation underscored the tangible impact of the three guidance methods on task efficiency and accuracy. These become integral to the user’s interaction experience during operations. For complex tasks, a pronounced execution time, considerable path deviation, and a heightened reliance on the operator’s proficiency and experience were observed. The key point indication method consistently showcased commendable performance across both task complexities, characterized by optimal execution times and minimal path deviations. Nonetheless, this method’s efficacy may be contingent upon the operator’s expertise. The real-time adjustment approach facilitates swift trajectory modifications during intricate tasks, leading to reduced execution times. Conversely, its application to simpler tasks results in elongated execution times and amplified path deviations.

From the vantage of task load, the study accentuated the disparities in users’ subjective experiences across the guidance methodologies. This segment offers a quantified perspective on the subjective experience, elucidating the repercussions of varied guidance methods on users. Complex tasks invariably necessitate substantial cognitive engagement, effort, and time and may induce heightened frustration. The key point indication method, given its consistent low cognitive demand and effort across both task types, emerges as apt for a spectrum of task difficulties. However, the operator’s expertise can potentially modulate its efficacy, particularly in determining key points. The real-time adjustment approach, in intricate tasks, demands relatively less cognitive strain and swiftly accommodates trajectory alterations. Yet, in simpler scenarios, it may induce greater cognitive strain and frustration.

The overarching evaluation of interaction experiences suggests that the key point indication method, with its reduced overall task load, is adept for tasks spanning varied complexities. For rudimentary tasks, direct trajectory planning remains preferable, while for more convoluted ones, real-time adjustment holds the upper hand. The choice among the three guidance methods should be predicated on the operator’s specific circumstances and requirements.

## 5. Conclusions and Outlook

This study was conducted within the context of digital twin robotic arm interactions. The guidance method for target motion input plays a pivotal role in human–machine interactions, prompting an exploration into the effects of different guidance methods on task completion and user interaction experiences. Both objective and subjective metrics quantified the user interaction experience, facilitating the assessment of the merits, demerits, and practical applications of direct trajectory planning, key point indication, and real-time adjustment.

Subjective task load and objective completion efficiency revealed that direct trajectory planning is apt for simple tasks typified by basic geometric shapes composed of linear segments. In contrast, the real-time adjustment method is tailored for complex tasks exemplified by irregular curves. Meanwhile, the key point indication method, with its reduced task load, is versatile enough for tasks across varying degrees of difficulty.

Supplementary to this study, interactive feedback experiments focusing on digital twin robotic arm interactions were executed. These furnished enhanced solutions, particularly in tactile and visual feedback domains. Future endeavors comprise designing a UR5 robotic arm digital twin interface prototype, drawing insights from preceding studies and experiments. Conclusively, target users will be solicited for user experience evaluations to corroborate the validity and efficacy of the research outcomes.

This investigation pioneers a novel research avenue for the application of digital twin technology. It furnishes a tangible blueprint for the interaction design of digital twin manipulators and offers invaluable insights for enhancing its interactive design user experience.

However, certain limitations punctuate this research. Firstly, the number of participants is a bit small. Secondly, the user experience evaluation leaned heavily on short-term experimental data. Prospective studies could benefit from long-term tracking to appraise the digital twin robotic arm interaction system’s performance more holistically in real-world applications. Additionally, the deployment of consumer-grade VR equipment for research inadvertently introduced discrepancies between the studied interaction methods and more organic interactions. The current device setup also presents complexities, potentially making it less user-friendly than conventional two-dimensional interfaces. Future endeavors ought to delve into alternative interaction modalities, such as voice commands and gesture recognition, contingent on advanced hardware systems, to engender a more immersive and intuitive interaction experience.

## Figures and Tables

**Table 1:** Summary of interaction demands.

Numbers	Interaction Demands
1	Task-specific interaction interface presentation
2	Logical interface layout
3	Robotic arm interactive guidance method
4	Reasonable interaction feedback forms
5	Effective interactive navigation
6	Optimal sensory experience
7	Visual presentation of information
8	Structured information architecture
9	Cognitive information management functions

**Table 2:** KANO evaluation result classification comparison table.

Demand Characteristic		Negative (If the Product Does not Meet the Required Characteristics, Your Evaluation Is)				
	Scale	I really like it	It should be	Indifferent	Settle for	I really do not like it
Positive (if the product has the required characteristics, your evaluation is)	I really like it	Q	A	A	A	O
	It should be	R	I	I	I	M
	Indifferent	R	I	I	I	M
	Settle for	R	I	I	I	M
	I really don’t like it	R	R	R	R	Q

**Table 3:** Summary of KANO model evaluation.

Demands Characteristic	A (Attractive Quality)	O (One-Dimensional Quality)	M (Must-be Quality)	I (Indifferent Quality)	R (Reverse Quality)	Q (Questionable Quality)	The Attributes KANO Leans towards	Better Value	Worse Value
1	0.36	0.21	0.27	0.08	0.05	0.03	A	0.61	−0.21
2	0.23	0.46	0.13	0.09	0.04	0.05	O	0.75	−0.73
3	0.12	0.30	0.48	0.10	0.00	0.00	M	0.42	−0.65
4	0.13	0.24	0.36	0.18	0.05	0.04	M	0.41	−0.56
5	0.48	0.21	0.15	0.09	0.03	0.04	A	0.73	−0.22
6	0.38	0.16	0.20	0.18	0.04	0.04	A	0.57	−0.37
7	0.09	0.54	0.27	0.10	0.00	0.00	O	0.63	−0.71
8	0.12	0.52	0.20	0.09	0.03	0.04	O	0.68	−0.61
9	0.05	0.23	0.50	0.13	0.04	0.05	M	0.32	−0.75

**Table 4:** Task completion time under simple tasks.

	Mean Value	Standard Deviation	Standard Error	95% Confidence Interval of the Mean	
				Lower Limit	Upper Limit
Direct trajectory planning	10.6387	1.9472	0.4359	9.7838	11.4936
Key point indication	11.8392	2.0459	0.4575	10.8946	12.7839
Real-time adjustment	13.1504	2.6433	0.5906	12.2065	14.0944

**Table 5:** Normality test of guidance method data.

	Guidance Method	Kolmogorov–Smirnov Test			Shapiro–Wilk Test		
		Statistics	Degree of freedom	Significance	Statistics	Degree of freedom	Significance
Task completion time	Direct trajectory planning	0.218	20	0.014	0.920	20	0.100
	Key point indication	0.139	20	0.200 * ^1^	0.938	20	0.219
	Real-time adjustment	0.218	20	0.014	0.903	20	0.048

^1^ * Indicates that this is already the lower limit of significance

**Table 6:** Homogeneity test of variance for task completion time.

		Levin Statistic	Number of Samples	Number of Variables	Significance
Task completion time	Based on mean	2.130	2	57	0.128
	Based on median	0.975	2	57	0.383
	Based on median and adjusted degrees of freedom	0.975	2	42.142	0.385
	Based on trimmed mean	1.881	2	57	0.162

**Table 7:** Testing of the inter-subject effects of guidance methods.

Source	Sum of Squares of Type III	Degree of Freedom	Mean Square	F	Significance
Calibration model	73.414a	2	36.707	8.319	0.001
Intercept	8769.751	1	8769.751	1987.587	0.000
Guidance method	73.414	2	36.707	8.319	0.001
Error	251.499	57	4.412		
Total	9094.664	60			
Corrected total	324.913	59			

**Table 8:** Task completion time under complex tasks.

	Mean Value	Standard Deviation	Standard Error	95% Confidence Interval of the Mean	
				Lower Limit	Upper Limit
Direct trajectory planning	22.3892	2.5294	1.1298	19.8789	24.8995
Key point indication	19.5934	3.6923	1.6517	16.8202	22.3666
Real-time adjustment	15.8468	2.1381	0.4789	14.9613	16.7322

**Table 9:** Normality test of task completion time.

	Guidance Method	Kolmogorov–Smirnov Test			Shapiro–Wilk Test		
		Statistics	Degree of freedom	Significance	Statistics	Degree of freedom	Significance
Task completion time	Direct trajectory planning	0.173	20	0.118	0.904	20	0.048
	Key point indication	0.084	20	0.200 * ^1^	0.981	20	0.951
	Real-time adjustment	0.105	20	0.200 * ^2^	0.967	20	0.701

^1 2^ * Indicates that this is already the lower limit of significance

**Table 10:** Robustness test for mean equality.

	Statistics	Number of Samples	Number of Variables	Significance
Welch	57.969	2	35.212	0.000

**Table 11:** Testing of inter-subject effects in task duration experiment.

Source	Sum of Squares of Type III	Degree of Freedom	Mean Square	F	Significance
Guidance method	134.925	2	67.463	10.942	0.000
Task complexity	1468.185	1	1468.185	238.125	0.000
Inter-subject effects	521.963	2	260.981	42.329	0.000

**Table 12:** Path deviation for simple tasks.

	Mean Value	Standard Deviation	Standard Error	95% Confidence Interval of the Mean	
				Lower Limit	Upper Limit
Direct trajectory planning	0.1083	0.0420	0.0094	0.0972	0.1193
Key point indication	0.1375	0.0594	0.0133	0.1247	0.1503
Real-time adjustment	0.1596	0.0469	0.0105	0.1461	0.1731

**Table 13:** One-way ANOVA under simple tasks.

	Sum of Squares	Degree of Freedom	Mean Square	F	Significance
Inter-group	583.474	2	291.737	36.840	0.000
Intra-group	451.379	57	7.919		
Total number	1034.853	59			

**Table 14:** Path deviations under complex tasks.

	Mean Value	Standard Deviation	Standard Error	95% Confidence Interval of the Mean	
				Lower Limit	Upper Limit
Direct trajectory planning	0.2005	0.0570	0.0128	0.1761	0.2249
Key point indication	0.1507	0.0389	0.0087	0.1297	0.1717
Real-time adjustment	0.1849	0.0367	0.0082	0.1614	0.2085

**Table 15:** One-way ANOVA of path deviation under complex tasks.

	Sum of Squares	Degree of Freedom	Mean Square	F	Significance
Inter-group	583.474	2	291.737	36.840	0.000
Intra-group	451.379	57	7.919		
Total number	1034.853	59			

**Table 16:** Testing of inter-subject effects in path deviations experiment.

Source	Sum of Squares of Type III	Degree of Freedom	Mean Square	F	Significance
Guidance method	0.019	2	0.009	4.405	0.014
Task complexity	0.030	1	0.030	14.248	0.000
Inter-subject effects	0.061	2	0.030	14.277	0.000

**Table 17:** Task load.

	Brain Power	Physical Strength	Time	Operational Performance	Effort	Frustration Level	Task Load
Guidance method	38.35	32.80	42.65	40.25	42.45	45.15	40.2750
Task complexity	26.15	31.10	35.60	31.45	37.20	28.75	31.7083
Guidance method Task complexity	32.50	31.75	40.30	39.70	48.50	50.60	40.5583

**Table 18:** Normality test of task load.

	Guidance Method	Kolmogorov–Smirnov Test			Shapiro–Wilk Test		
		Statistics	Degree of freedom	Significance	Statistics	Degree of freedom	Significance
Task load	Direct trajectory planning	0.092	20	0.200 * ^1^	0.977	20	0.889
	Key point indication	0.153	20	0.200 * ^2^	0.959	20	0.525
	Real time adjustment	0.249	20	0.002	0.846	20	0.005

^1 2^ * Indicates that this is already the lower limit of significance.

**Table 19:** Homogeneity test of variance for task load.

		Levin Statistic	Number of Samples	Number of Variables	Significance
Task load	Based on mean	0.720	2	57	0.491
	Based on median	0.335	2	57	0.716
	Based on median and adjusted degrees of freedom	0.335	2	47.772	0.717
	Based on trimmed mean	0.698	2	57	0.502

**Table 20:** One-way ANOVA.

	Sum of Squares	Degree of Freedom	Mean Square	F	Significance
Inter-group	743.464	2	371.732	122.020	0.000
Intra-group	173.650	57	3.046		
Total number	917.114	59			

**Figure A1:**
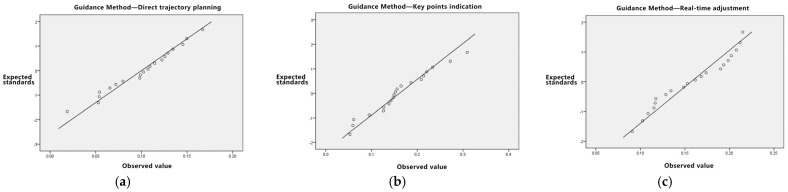
Standard Q-Q diagram for path deviation. (**a**) Direct trajectory planning. (**b**) Key points indication. (**c**) Real-time adjustment.

## Data Availability

The datasets used and/or analyzed during the current study are available from the corresponding author upon reasonable request.

## Appendix A

The KANO model survey questionnaire is shown in Table A1.

**Table A1:** Translated version of Table A1.

	I Really Like It	It Should Be	Indifferent	Settle for	I Really Do not Like It
If the product has interactive interface representations for different tasks	○	○	○	○	○
If the product does not have interactive interface representations for different tasks	○	○	○	○	○
If the product has a reasonable interface layout	○	○	○	○	○
If the product does not have a reasonable interface layout	○	○	○	○	○
If the product has a robotic arm interactive guidance method	○	○	○	○	○
If the product does not have a robotic arm interactive guidance method	○	○	○	○	○
If the product has a reasonable form of robotic arm interaction feedback	○	○	○	○	○
If the product does not have a reasonable form of robotic arm interaction feedback	○	○	○	○	○
If the product has good interactive navigation	○	○	○	○	○
If the product does not have good interactive navigation	○	○	○	○	○
If the product has a good sensory experience	○	○	○	○	○
If the product does not have a good sensory experience	○	○	○	○	○
If the product has a visual presentation of information	○	○	○	○	○
If the product does not have a visual presentation of information	○	○	○	○	○
If the product has a reasonable information architecture	○	○	○	○	○
If the product does not have a reasonable information architecture	○	○	○	○	○
If the product has cognitive information management capabilities	○	○	○	○	○
If the product does not have cognitive information management functions	○	○	○	○	○

## Appendix B

Some experimental data are as follows. The standard Q-Q diagram for path deviation is shown in Figure A1. The homogeneity of variance test for path deviation is shown in Table A2.

**Table A2:** Homogeneity of variance test for path deviation.

		Levin Statistic	Number of Samples	Number of Variables	Significance
Path deviation	Based on mean	2.942	2	57	0.061
	Based on median	2.352	2	57	0.104
	Based on median and adjusted degrees of freedom	2.352	2	35.233	0.110
	Based on trimmed mean	2.764	2	57	0.071
